# Analyzing nonlinear contributions from climate change and anthropogenic activity to the normalized difference vegetation index across China using a locally weighted regression approach

**DOI:** 10.1016/j.heliyon.2023.e16694

**Published:** 2023-05-25

**Authors:** Chenhua Shen, Rui Wu

**Affiliations:** a College of Geographical Science, Nanjing Normal University, Nanjing, 210046, China; b Key Laboratory of Virtual Geographic Environment of Ministry of Education, Nanjing, 210046, China; c Jiangsu Center for Collaborative Innovation in Geographical Information Resource, Nanjing, 210046, China

**Keywords:** Nonlinear contribution, Meteorological factors, Anthropogenic activity, NDVI, Changing trend

## Abstract

Nonlinear contributions from climate change and anthropogenic activity to the Normalized Difference Vegetation Index (NDVI) are analyzed to better understand the mechanisms underlying the nonlinear response of vegetation growth. In this study, it was hypothesized that NDVI dynamics on a nonlinear trajectory could track fluctuations of climate change and anthropogenic activity. Contributions from climate change and anthropogenic activity to NDVI were quantified using a locally weighted regression approach based on monthly timescale datasets. The findings showed that: 1) Vegetation cover fluctuated and increased in 81% of regions in China from 2000 to 2019. 2) The average predicted nonlinear contribution (APNC) of anthropogenic activity to NDVI was positive in China. The temperature APNC was positive in most of China but negative in Yunnan, where high temperatures and asynchronous temporal changes in temperature and NDVI were observed. The precipitation APNC was positive in the north of the Yangtze River, where precipitation is insufficient; but negative in South China, where precipitation is plentiful. Anthropogenic activity had the highest magnitude among the three nonlinear contributions, followed by temperature and precipitation. 3) The regions with contribution rates of anthropogenic activity greater than 80% were mainly distributed in the central Loess Plateau, North China Plain, and South China, while the areas with contribution rates of climate change greater than 80% were mainly concentrated in the northeastern QTP, Yunnan, and Northeast China. 4) The high temperature, drought, and asynchronous temporal changes in temperature, precipitation, and NDVI caused the negative average of changing trends in the predicted nonlinear contribution (PNC) of climate change to NDVI. Deforestation, land cover change, and grazing/fencing led to the negative average of changing trends in PNC from anthropogenic activity. These findings deepen our understanding of the mechanisms underlying the nonlinear responses of vegetation growth to climate change and anthropogenic activity.

## 1 Introduction

Vegetation is an essential component of global terrestrial ecosystems and is responsible for material circulation, energy flow, and information transmission through plant hormones, odors, and specific substances [1]. Previous studies have demonstrated that climate change and anthropogenic activity are the primary driving factors for variation in vegetation cover in terrestrial ecosystems, according to linear analytical methods [2,3]. Given that terrestrial ecosystems are susceptible to climate change and anthropogenic activity, a better understanding of the nonlinear responses of vegetation growth to climate change and anthropogenic activity could aid in achieving future sustainable goals for ecosystem development.

The status of vegetation growth is a comprehensive response to climate change. The Normalized Difference Vegetation Index (NDVI) is commonly used to effectively monitor vegetation growth and determine vegetation feedback at global and regional scales using data on vegetation greenness, according to red and near-infrared reflection datasets [[4], [5], [6]]. Temperature and precipitation are key variables that reflect the physical traits of climate environment and have dual effects on vegetation growth. In the central and southeastern regions of the Loess Plateau in China, climate warming over the past 30 years has promoted vegetation restoration but has had an adverse effect on vegetation restoration in northwestern China [7,8]. Global precipitation deficiency and high atmospheric temperatures led to drought hazards in the 1980s, reducing vegetation cover in certain areas with high latitudes, particularly in the northern hemisphere [9,10]. However, excessive precipitation in South China did not promote robust vegetation growth.

Anthropogenic activities also affect vegetation growth in two ways. Vegetation cover has been substantially reduced because land that was previously used for agriculture, woodland, wetland, and grassland is occupied by urban expansion, infrastructure construction, mining, and deforestation activities [7]. In contrast, vegetation cover has increased due to various anthropogenic activities, such as returning farmland to forest and grassland, implementing projects to construct ecological forests and nature reserves, and afforestation [11].

Anthropogenic activities interact with climate change through various hierarchical interconnections, influencing the structure and function of terrestrial ecosystems [12]. Directly measuring the relative contributions of anthropogenic activities and climate change to NDVI has proven challenging. Previous studies have used several direct and indirect approaches and estimation models, such as manipulation experiments, field observations, monitoring by remote sensing, and mechanistic and empirical modeling [13,14], to identify interactive effects of climatic element and anthropogenic activities on vegetation dynamics. Field experimentation [15] was relatively precise among these methods. However, policymakers have been reluctant to accept the results because of the gap between practical applications and ideal laboratory experimental conditions.

Results acquired by remote sensing are generally closer to practical applications. Based on remote sensing data, various statistical techniques, such as trend analyses of residuals [16,17], correlation metrics [18], and principal component analyses [19], have been used to determine the relative sensitivity of vegetation indices to climate change and anthropogenic activity. Residual trend modeling (RESTREND) [16] is used frequently [20], in which the slopes of two time series can be used to distinguish the relative importance between climate change and anthropogenic activity for NDVI dynamics. To accomplish this, the slopes of the two decomposed components are directly compared after the original year-NDVI time series is decomposed into two parts: one part is attributed to climatic factors and the other to non-climatic factors that can be tentatively interpreted as anthropogenic activities. Although RESTREND analysis can provide information on practical implications for ecosystem management and conservation, it is frequently criticized because the climate-vegetation linear relationship is empirically assumed. A global linear model cannot adequately capture the nonlinearity and complexity of ecosystem responses to an external force [[21], [22], [23]]. The traditional regression method (global linear regression) often works based on the assumption that the vegetation growth is stable over time [21]. When regression coefficients are estimated by the ordinary least squares (OLS) method, they are unreliable, unstable, or even spurious [24] if the residual error of the regression model does not pass the normal distribution test or is characterized by autocorrelation or heteroscedasticity. Therefore, in the RESTREND method, when the residual error passes the normal distribution test, the regression coefficients are reliable. However, anthropogenic activities are not detected because the residual error contains no information. In contrast, the regression coefficients are unstable or uncertain if the residual error fails to pass the normal distribution test. Under this circumstance, anthropogenic activities detected by the residual error are uncertain or unstable.

The vegetation growth varies unevenly over time due to seasonal change [25], i.e., the responses of vegetation growth to climate change and anthropogenic activity are nonlinear [21]. Three mechanisms cause this: 1) asynchronous temporal changes [26] in vegetation growth, temperature, and precipitation; 2) low and high threshold values for temperature or precipitation related to vegetation growth [27]; and 3) spatial heterogeneities of temperature, precipitation, or NDVI [25]. In this context, a method suitable for spatio-temporally local circumstances was necessary.

Recently, nonlinear and gradual changes in secular trends in NDVI have gained more attention. Piecewise linear regression [25], Theil–Sen estimation [28], Mann–Kendall test, and polynomial fit [29] methods, which depend on nonlinear regression models, have been used to estimate nonlinear trends. However, these methods neglect seasonal changes in vegetation growth, particularly the changes in the contribution of temperature and precipitation during vegetation growing period to NDVI. The assumption of linearity may oversimplify the evolutionary processes in vegetation trends.

In this study, an alternative method, the locally weighted regression model (LWLR), was introduced to explore the nonlinear contributions of climate change and anthropogenic activity to NDVI. This technique can identify the relative roles of two contributions in NDVI dynamics depending on the changing trends in the nonlinear contributions of climate change and anthropogenic activity over time [11,[30], [31], [32]]. The two primary characteristics of the LWLR model are as follows: 1) the coefficients of the LWLR model are permitted to vary with time, and 2) the weighting function is asymmetrical, which has different bandwidths on the left and right sides. The asymmetry of the weighting function describes interactions of left and right sides between NDVI and climate change with different mechanisms. This nonlinear modeling provides excellent prediction precision because the model is better attuned to local circumstances over time.

The aims of this study were to: 1) quantify the nonlinear contributions from climate change and anthropogenic activity to NDVI by applying the LWLR method and to analyze their changing trends over time; 2) analyze the relative importance of climatic factors and anthropogenic activity; 3) obtain the spatial distribution of nonlinear contributions from climate change and anthropogenic activity to NDVI at the national-level scale; and 4) explore the mechanisms of changing trends in nonlinear contributions.

The results of this study provide nonlinear and non-stationary insights into the regulatory mechanisms and driving factors of vegetation growth. Furthermore, the findings support the science-based theoretical guidance for ecological management and sustainable development in China.

## 2 Methods

### 2.1 Estimating the nonlinear contributions of climate change and anthropogenic activity to NDVI

As vegetation growth is affected by climatic factors (e.g., temperature and precipitation) and anthropogenic activities, NDVI is a combination of temperature and precipitation at a specific grid point [10] as Eq. (1):

(1) *N*(*t*) = f(*T*(*t*)) + g(*P*(*t*)) + β_0_ + *ε*(*t*), (1 ≤ *t* ≤ *M*)

where *N*, *T*, *P*, and *ε* represent the time series of NDVI, temperature, precipitation, and residual error, respectively; β_0_ is a constant intercept term. Herein, *t* is the temporal index, i.e., the month from January 2000 to December 2019 in this study. *M* is the length of the time series. f (*T*(*t*)) and g (*P*(*t*)) are two functions referring to contributions from temperature and precipitation to NDVI, respectively. They are usually expressed by quadratic functions as Eq. (2) [10]:

(2) $$f\left(T\left(t\right)\right)=\beta_{1}T\left(t\right)+\beta_{2}T^{2}\left(t\right),and\;g\left(P\left(t\right)\right)=\beta_{3}P\left(t\right)+\beta_{4}P^{2}\left(t\right)$$

When Eq. (2) is substituted into Eq. (1), Eq. (1) is rewritten as Eq. (3):

(3) $$N\left(t\right)=\beta_{1}T\left(t\right)+\beta_{2}T^{2}\left(t\right)+\beta_{3}P\left(t\right)+\beta_{4}P^{2}\left(t\right)+\beta_{0}+\varepsilon \left(t\right)$$

where β_0_ – β_4_ are constant regression coefficients. Eq. (3) is termed a global linear regression model when *N*(*t*) is defined as a dependent variable, and *T*(*t*), *P*(*t*), *T*^2^(*t*), and *P*^2^(*t*) are defined as independent variables. The OLS method normally estimates all constant regression coefficients in Eq. (3).

The global determinable coefficient of Eq. (3), denoted as $R_{g}^{2}$, is:

(4) $$R_{g}^{2}=\frac{\sum_{t}{\left[\hat{N}\left(t\right)-\bar{N\left(t\right)}\right]}^{2}}{\sum_{t}{\left[N\left(t\right)-\bar{N\left(t\right)}\right]}^{2}}=1-\frac{\sum_{t}{\left[N\left(t\right)-\hat{N}\left(t\right)\right]}^{2}}{\sum_{t}{\left[N\left(t\right)-\bar{N\left(t\right)}\right]}^{2}}$$

Eq. (4) represents the explanatory ability of all independent variables for a dependent variable. The greater the $R_{g}^{2}$ value, the stronger the explanatory ability. Since correlations between temperature, precipitation, and NDVI are nonlinear, temperature or precipitation might have a unique explanatory ability for NDVI at different time points. Eq. (3), therefore, has a clear limitation as a global linear regression model. To overcome this limitation, the LWLR model, which is more appropriate in this case than a global linear regression model, was introduced because the basis for the LWLR model is that the coefficients at each time point are locally estimated depending on the temporal distance-weighted sub-sampling undertaken at neighboring time points.

Eq. (3) is then improved as Eq. (5), as follows:

(5) $$N\left(t\right)=\beta_{1}\left(t\right)T\left(t\right)+\beta_{2}\left(t\right)T^{2}\left(t\right)+\beta_{3}\left(t\right)P\left(t\right)+\beta_{4}\left(t\right)P^{2}\left(t\right)+\beta_{0}\left(t\right)+\varepsilon \left(t\right)$$

For convenience, *x*_1_(*t*) = *T*(*t*), *x*_2_(*t*) = *T*^2^(*t*), *x*_3_(*t*) = *P*(*t*), and *x*_4_(*t*) = *P*^2^(*t*) are denoted. Eq. (5) is then formally written as Eq. (6):

(6) $$N\left(t\right)=\beta_{0}\left(t\right)+\sum_{i=1}^{i=4}\beta_{i}\left(t\right)x_{i}\left(t\right)+\varepsilon \left(t\right)$$

The parameters *β*_0_(*t*) – *β*_4_(*t*) in Eq. (6) are the time-varying regression coefficients of the *t*th time point for independent variables, including *T*(*t*), *P*(*t*), *T*^2^(*t*), and *P*^2^(*t*), reflecting the respective contribution ratios of temperature and precipitation to NDVI at time point *t*. *β*_1_(*t*) *– β*_4_(*t*) are implicit functions that could capture the temporal non-stationary contributions of temperature and precipitation [33]. The intercept term *β*_0_(*t*) is thereby preliminarily interpreted as a variable associated with anthropogenic activity to a certain extent because it is attributed to non-climatic factors.

To estimate *β*_0_(*t*) – *β*_4_(*t*), the LWLR model that takes the version of the OLS model is represented with the fixed time point *t* as follows:

(7) $${Q\left(t\right)=\mathrm{argmin}\;(\sum }_{j=1}^{j=M}W\left(j,t\right){\left[N\left(j\right)-\beta_{0}\left(t\right)-\sum_{i=1}^{i=4}\beta_{i}\left(t\right)x_{i}\left(j\right)\right]}^{2})$$

where W (*j*, *t*) is a weighting function. The estimator which uses matrix representation can be expressed by Eq. (8) as follows:

(8) $$\hat{\beta }\left(t\right)={\left(X^{T}W\left(t\right)X\right)}^{-1}\left(X^{T}W\left(t\right)N\right)$$

Given that the time series of temperature and precipitation often have autocorrelation [26], the residual error *ε*(*t*) in Eq. (6) is likely to be auto-correlated on occasion. Under this circumstance, the estimated time-varying regression coefficients ${\hat{\beta }}_{0}\left(t\right)-{\hat{\beta }}_{4}\left(t\right)$ will likely be uncertain or unstable when Eq. (8) is used. Therefore, the weighted Durbin**–**Watson coefficient should be used to measure whether the residual error *ε*(*t*) is auto-correlated [34], and the Cochrane–Orcutt iterative method [35] is used to remove or reduce the autocorrelation degree of residual error.

The determinable coefficient of the LWLR model (Eq. (6)) is defined as

(9) $$R_{l}^{2}=\frac{\sum_{t}{\left[\hat{N}\left(t\right)-\bar{N\left(t\right)}\right]}^{2}}{\sum_{t}{\left[N\left(t\right)-\bar{N\left(t\right)}\right]}^{2}}=\frac{\sum_{t}{\left[{\hat{\beta }}_{0}\left(t\right)+\sum_{i=1}^{i=4}{\hat{\beta }}_{i}\left(t\right)x_{i}\left(t\right)-\bar{N\left(t\right)}\right]}^{2}}{\sum_{t}{\left[N\left(t\right)-\bar{N\left(t\right)}\right]}^{2}}$$

According to Eqs. (4), (9)), the ratio is defined as:

(10) $$R_{\mathrm{lg}}=R_{l}^{2}/R_{g}^{2}$$

$R_{\mathrm{lg}}$ >1 indicates that the explanatory ability of all independent variables for temperature and precipitation for the dependent variable in Eq. (6) is stronger than that of a global linear regression model (Eq. (3)).

A weighting function is usually established with a distance decay parameter. Given that *T* (*t* − *n*) has an effect on *N*(*t*) when *n* > 0, *T* (*t* + *n*) has few effects on *N*(*t*) because the future temperature has a minimal impact on current vegetation growth. The vegetation's growth rhythm determines this. For temperature on the left and right sides at the *t*th time point, their degrees of cross-correlation between with NDVI differ. Similar results are valid for the precipitation. Therefore, the asymmetrical functional structure of W (*j*, *t*) against the *j*th time point around time *t* is required.

Gaussian-based kernels are frequently used as weighting functions. The fixed Gaussian-based kernel function with dual bandwidth parameters is improved as follows:

(11) $$W\left(j,t\right)=\left\{ \begin{array}{l} \exp\;{(-\frac{1}{2}\left(\frac{j-t}{h_{1}}\right)}^{2})\;for\;j<t\\ \exp\;{(-\frac{1}{2}\left(\frac{j-t}{h_{2}}\right)}^{2})\;for\;j\geq t \end{array}\right.$$

where *h*_1_ and *h*_2_ are two non-negative bandwidth parameters that generate an attenuation effect related to the temporal distance between the *j*th and *t*th time points. *R*_h_ = *h*_2_/*h*_1_ is defined. *R*_h_ > 1 indicates that the cases of NDVI temporally lagging behind a meteorological element are dominant, whereas those of a meteorological element temporally lagging behind NDVI are dominant for *R*_h_ < 1 [36].

Adaptive kernels with adaptive bandwidths ensure adequate local information for each calibration [33]. Cross-validation (CV) is an iterative process that searches for the optimal kernel bandwidth that minimizes the prediction error of all *N*(*t*) using a subset of the data for prediction [37]. The optimal kernel bandwidth is estimated in CV by finding that it minimizes the root mean squared prediction error:

(12) $$CV\left(h\right)=\mathrm{argmin}{\sum_{t}\left(N\left(t\right)-{\hat{N}}_{\neq t}\left(t\right)\right)}^{2}$$

where ${\hat{N}}_{\neq t}\left(t\right)$ is the *t*th predicted value with the *t*th calibration time point left out of the estimation dataset. This method is called “leave-one-out” CV because one observation data point is removed from the estimation dataset for each local model when estimating the optimal kernel bandwidths. The *t*th time point is removed when estimating ${\hat{N}}_{\neq t}\left(t\right)$ to avoid a perfect estimation.

${\hat{\beta }}_{0}\left(t\right)-{\hat{\beta }}_{4}\left(t\right)$ are the estimated time-varying regression coefficients of *β*_0_(*t*) – *β*_4_(*t*) according to Eqs. (7), (8)). $\hat{f}\left(T\left(t\right)\right)={\hat{\beta }}_{1}\left(t\right)T\left(t\right)+{\hat{\beta }}_{2}\left(t\right)T^{2}\left(t\right)$ and $\hat{g}\left(P\left(t\right)\right)={\hat{\beta }}_{3}\left(t\right)P\left(t\right)+{\hat{\beta }}_{4}\left(t\right)P^{2}\left(t\right)$ are predicated and termed the predicated nonlinear contributions (PNC) from temperature and precipitation to NDVI. ${\hat{\beta }}_{0}\left(t\right)$ is termed the PNC from anthropogenic activity to NDVI.

(13) $$\bar{f}=\left\langle \hat{f}\left(T\left(t\right)\right)\right\rangle =\frac{1}{M}\sum_{t=1}^{t=M}\hat{f}\left(T\left(t\right)\right)$$

(14) $$\bar{g}=\left\langle \hat{g}\left(P\left(t\right)\right)\right\rangle =\frac{1}{M}\sum_{t=1}^{t=M}\hat{g}\left(P\left(t\right)\right)$$

(15) $${\bar{\beta }}_{0}=\left\langle {\hat{\beta }}_{0}\left(t\right)\right\rangle =\frac{1}{M}\sum_{t=1}^{t=M}{\hat{\beta }}_{0}\left(t\right)$$

Eqs. (13), (14), (15)) represent the average of the predicted nonlinear contribution (APNC) of temperature, precipitation, and anthropogenic activity over the entire time series. When $\bar{f}+\bar{g}>{\bar{\beta }}_{0}$, the APNC from climate change is dominant, otherwise, that from anthropogenic activity is dominant. The APNC from climate change is positive when $\bar{f}+\bar{g}>0$, indicating that the climate environment is suitable for vegetation growth. The APNC from anthropogenic activity is negative when ${\bar{\beta }}_{0}<0$, suggesting that anthropogenic activities hinder or damage vegetation growth. The relationship between $\bar{f}+\bar{g}$ and ${\bar{\beta }}_{0}$ discloses nonlinear contributions of climate change and anthropogenic activity to NDVI.

### 2.2 Identifying the relative roles of climate and anthropogenic activity in NDVI dynamics

The predicated nonlinear contributions $\hat{f}\left(T\left(t\right)\right)$, $\hat{g}\left(P\left(t\right)\right)$, and ${\hat{\beta }}_{0}\left(t\right)$ vary over time. For simplicity, $\hat{F_{G}}\left(t\right)$ = $\hat{f}\left(T\left(t\right)\right)+\hat{g}\left(P\left(t\right)\right)$ is defined. $\hat{F_{G}}\left(t\right)$ is decomposed into the trend, seasonal, and remainder components using the seasonal trend-decomposition procedure (STL) [38] to eliminate the seasonal effect. The decomposed trend term of $\hat{F_{G}}\left(t\right)$ is denoted as ${\hat{F_{G}}}^{T}\left(t\right)$. Given that ${\hat{\beta }}_{0}\left(t\right)$ and ${\hat{F_{G}}}^{T}\left(t\right)$ vary over time, they are fitted by a cubic function. The two parameters *α* and *α*_0_ are defined as follows:

(16) $$\alpha =\left\langle \frac{d{\hat{F_{G}}}^{T}\left(t\right)}{dt}\right\rangle$$

(17) $$\alpha_{0}=\left\langle \frac{d{\hat{\beta }}_{0}\left(t\right)}{dt}\right\rangle$$

Eqs. (16), (17)) represents the average changing trends in ${\hat{F_{G}}}^{T}\left(t\right)$ and $\hat{\beta_{0}}\left(t\right)$. When *α>*0, ${\hat{F_{G}}}^{T}\left(t\right)$ approximately increases with time, indicating that either vegetation cover is increasing or vegetation growth conditions are improving due to climate change. In contrast, ${\hat{F_{G}}}^{T}\left(t\right)$ roughly decreases when *α<*0, suggesting that either the vegetation is browning or the land is degraded. Similar results are valid for the average *α*_0_. The relationship between *α* and *α*_0_ illustrates the roles of climate change and anthropogenic activity in NDVI dynamics [16]. We determined the relative roles of climate change and anthropogenic activity according to the judging criteria from Jin et al. [7].

To analyze the ingredient structure of *α* in Eq. (16), the second-order terms for temperature and precipitation on the right side of Eq. (5) are disregarded because they are relatively small. The ingredient structure of *α* is, therefore, given in terms of the one-order derivative of time in Eq. (18):

(18) $$\alpha =\left\langle \frac{d{\hat{F_{G}}}^{T}\left(t\right)}{dt}\right\rangle \approx \left\langle T\left(t\right)\frac{d{\hat{\beta }}_{1}\left(t\right)}{dt}+{\hat{\beta }}_{1}\left(t\right)\frac{dT\left(t\right)}{dt}+P\left(t\right)\frac{d{\hat{\beta }}_{3}\left(t\right)}{dt}+{\hat{\beta }}_{3}\left(t\right)\frac{dP\left(t\right)}{dt}\right\rangle =\left\langle \left(T\left(t\right)\frac{d{\hat{\beta }}_{1}\left(t\right)}{dT\left(t\right)}+{\hat{\beta }}_{1}\left(t\right)\right)\frac{dT\left(t\right)}{dt}+\left(P\left(t\right)\frac{d{\hat{\beta }}_{3}\left(t\right)}{dP\left(t\right)}+{\hat{\beta }}_{3}\left(t\right)\right)\frac{dP\left(t\right)}{dt}\right\rangle$$

According to Eq. (18), the time-varying regression coefficients ${\hat{\beta }}_{1}\left(t\right)$ and ${\hat{\beta }}_{3}\left(t\right)$, their changing trends $\frac{d{\hat{\beta }}_{1}\left(t\right)}{dt}$ and $\frac{d{\hat{\beta }}_{3}\left(t\right)}{dt}$, temperature *T*(*t*), and precipitation *P*(*t*) influence the ingredient structure of *α.* The parameter *α* still reveals the one-order time-lagging correlations between NDVI and temperature and between NDVI and precipitation.

Eqs. (19), (20)) show the fitted polynomial functions (e.g., a cubic function) of ${\hat{\beta }}_{1}\left(t\right)$ and ${\hat{\beta }}_{3}\left(t\right)$:

(19) $${\hat{\beta }}_{1}\left(t\right)=m_{3}t^{3}+m_{2}t^{2}+m_{1}t+m_{0}+\varepsilon_{1}\;\left(t\right)$$

(20) $${\hat{\beta }}_{3}\left(t\right)=n_{3}t^{3}+n_{2}t^{2}+n_{1}t+n_{0}+\varepsilon_{3}\;\left(t\right)$$

where m_0_ – m_3_ and n_0_ – n_3_ are the fitting coefficients estimated using the OLS method.

(21) $$\alpha_{1}=\left\langle \frac{d{\hat{\beta }}_{1}\left(t\right)}{dt}\right\rangle =\frac{1}{M}\sum_{t=1}^{t=M}\left(3m_{3}t^{2}+2m_{2}t+m_{1}\right)$$

(22) $$\alpha_{3}=\left\langle \frac{d{\hat{\beta }}_{3}\left(t\right)}{dt}\right\rangle =\frac{1}{M}\sum_{t=1}^{t=M}\left({3n}_{3}t^{2}+2n_{2}t+n_{1}\right)$$

The parameters *α*_1_ and *α*_3_ represent the average changing trend in ${\hat{\beta }}_{1}\left(t\right)$ and ${\hat{\beta }}_{3}\left(t\right)$.

### 2.3 Testing the normal probability distribution of residual error

The probability distribution of residual error *ε*(*t*) is tested to evaluate the reliability of the LWLR model. If the residual error *ε*(*t*) passes the normal probability distribution test, the LWLR model is considered reliable. Next, the theoretical probability density function of residual error *ε*(*t*) is calculated using the normal probability density function N (*u*, *σ*), whereas its practical probability density function is estimated by applying Gaussian kernel density estimation techniques [39]. Finally, the absolute value of the maximum difference between two probability functions, ΔP, is calculated. When ΔP is less than a threshold value *τ*, the residual error *ε*(*t*) is considered to pass the normal probability distribution testing; otherwise, it fails.

The threshold value *τ* in this study was determined using the Monte Carlo simulation method by artificially generating 10,000 datasets with a normal probability density function of N (*u* = 0, *σ* = 1) using Matlab R13 (https://www.mathworks.com/products/matlab. html), and the ΔP for each dataset was calculated. The average and variance of ΔP were then estimated across all the datasets. Finally, the threshold value *τ* was determined according to the average ΔP.

## 3 Study area and data sources

### 3.1 Study area

The study area encompassed the entire territory of China from 3° 51′ to 53° 33′ N and 73° 33′ to 135° 5′ E. A total of 15,248 grid points with spatial resolutions of 0.25° × 0.25° were created. The central coordinate for each grid point was used to create a square with sides 0.25° long. Taiwan, Hong Kong, and Macao were not included because of the absence of data sources. Regarding temperature zone division, China is divided into tropical, subtropical, warm temperate, medium temperate, cold temperate, and Qinghai-Tibet Plateau regions [40]. Due to the substantial differences in distance from the sea in different regions, combined with diverse terrains, mountain orientations, and atmospheric circulation, China has diverse climate environments [41]. Differences in heat and humidity exist even within the same climate type. Complex landform types, diverse climate environments, and the impact of anthropogenic activities have resulted in remarkable differences in vegetation cover in different regions of China. Therefore, China is an ideal study area.

### 3.2 Data sources

Currently, the most commonly used NDVI data include SPOTVGT NDVI, AVHRR NDVI, and MODIS NDVI. These three sources differ in sensors, spectral response functions, correction and data processing methods, and spatial-temporal resolution. MODIS NDVI was selected for this study because it has a high spatial resolution.

The MOD13A3 NDVI dataset (level-3 product, monthly, 1 km) derived from the Terra MODIS instrument was processed to assess vegetation growth across China from 2000 to 2019 (https://search.earthdata.nasa.gov/search). Geometric, radiometric, and atmospheric corrections were made to the MOD13A3 NDVI dataset [42]. The maximum value composite method minimizes the cloud-cover, scan-angle, and solar-zenith-angle effects [43]. The corrected NDVI images were mosaiced and re-projected from the Sinusoidal to the Albers Conical Equal Area projection using the MODIS Re-projection Tool. The NDVI value was extracted according to the central coordinates of each grid point.

Monthly synchronous data records of temperature and precipitation with NDVI were retrieved from CN05.1 (ftp://159.226.119.13, accessed on July/30/2022). CN05.1 daily grid observation datasets with a spatial resolution of 0.25° × 0.25°, spanning January 1961 to December 2020, were produced through interpolation and combination. Based on observation data from more than 2400 national-level meteorological stations in China [44,45], the meteorological datasets CN05.1 were created using two interpolating techniques: a partial thin plate spline method [46] and an angle-distance weight method [44]. The extracted NDVI, data records of temperature and precipitation, and the results from this study have an exact spatial resolution of 0.25° × 0.25°.

## 4 Results

When the average NDVI throughout the study period was **≤**0.15 at a particular grid point, it was classified as having no vegetation. These points were excluded from the analysis. Consequently, 4913 grid points (approximately 32.2% of the total) were excluded. The blank areas in Fig. 2, Fig. 3, Fig. 4 represent the excluded grid points, which were predominantly located in the desertified regions of Northwest China and areas covered with extensive water bodies (e.g., Yangtze River, Dongting Lake, and Taihu Lake). This study did not provide information regarding the nonlinear contributions at these grid points. As a result, Eq. (1) could be fitted for 10,335 grid points, which comprised nearly 67.8% of all grid points.

Using weighted variance inflation factors (VIF) [47], the respective collinearity between *T*(*t*) and *P*(*t*), *T*(*t*) and *P*^2^(*t*), and *T*^2^(*t*) and *P*(*t*) in Eq. (5) was diagnosed for each grid point. For approximately 99.5% of all grid points, the maximum VIF was less than 4. The result indicates that the collinearity in Eq. (5) was relatively weak. Additionally, the weighted Durbin–Watson coefficients [34] of the residual error *ε*(*t*) were estimated, and they ranged from 1.70 to 2.30 for approximately 94.0% of all grid points with a 95% confidence level, indicating that the residual error had a weak autocorrelation.

The ratio $R_{\mathrm{lg}}=R_{l}^{2}/R_{g}^{2}$ was calculated using Eq. (4) and 9–10. Table 1 shows the fraction of all grid points according to the level. The grid points with *R*_lg_>1.1 accounted for approximately 63.5%. The grid points with *R*_lg_>1.5 were predominantly distributed in South China, including Fujian, Guangdong, Guangxi, and Yunnan. This finding implies that the temperature and precipitation in the LWLR model had a better explanatory ability for NDVI than those in the global linear regression model (Eq. (1)). The NDVI variance is more effectively interpreted with temperature and precipitation, making the LWLR model more helpful than the global linear regression model in this context.

**Table 1:** Number of grid points and their percentage of different levels of R_lg_.

Ratio/*R*_lg_	1.00–1.10	1.11–1.20	1.21–1.30	1.31–1.40	1.41–1.50	>1.50
Number of grid points (%)	3774 (36.5)	2132 (20.6)	1249 (12.1)	759 (7.3)	480 (4.6)	1941 (18.9)

The residual error *ε*(*t*) was estimated using Eq. (5). The ΔP between the theoretical and practical probability functions was calculated using the approach detailed in Section 2.3. The threshold value *τ* was determined using the Monte Carlo simulation method and was set to 0.04. The criterion for this threshold value was substantially tighter than that of the Kolmogorov–Smirnov test [48]. This demonstrates that the LWLR model was reliable. The grid points with ΔP < 0.04 and ΔP∈(0.04, 0.06) accounted for 52.0% and 25.0% of all grid points, respectively.

### 4.1 Time-varying regression coefficients and contributions of the typical grid point

**Fig. 1:**
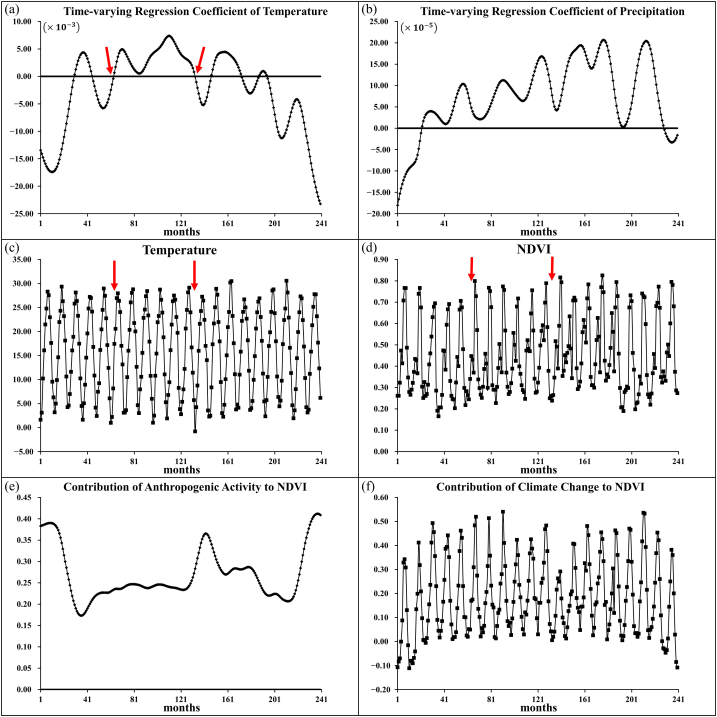
Time-varying regression coefficients (Panels a and b) of temperature and precipitation, temperature time series (Panel c), NDVI time series (Panel d), contribution of climate (Panel e), and contribution of anthropogenic activity (Panel f) against time for a typical grid point.

**Fig. 2:**
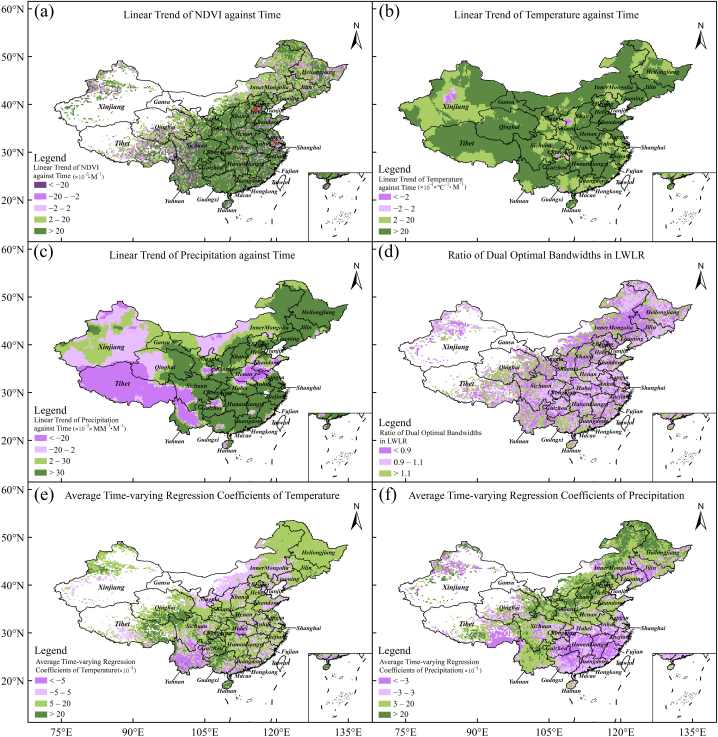
Basic statistical parameter in LWLR. Panel (a) for monthly slope of NDVI time series. Panels (b and c) for monthly slope of temperature and precipitation time series, respectively. Panel (d) for the ratio of dual optimal bandwidths. Panels (e and f) for the averages of time-varying regression coefficients of temperature and precipitation over time, respectively.

Fig. 1a–f shows the time-varying regression coefficients ${\hat{\beta }}_{1}\left(t\right)$ and ${\hat{\beta }}_{3}\left(t\right)$, the time series of temperature, time series of NDVI, $\hat{\beta_{0}}\left(t\right)$ and ${\hat{F_{G}}}^{T}\left(t\right)$, respectively, of the typical grid point (see the red dot around marked “A” in Fig. 2a). The positive time-varying regression coefficient of temperature, ${\hat{\beta }}_{1}\left(t\right)$, falling between two red arrows (Fig. 1a), was reasonable because the temperature between the two red arrows in Fig. 1c was higher than that on the left side of the red arrow. The NDVI value falling between the two red arrows in Fig. 1d was greater than that on the left side of the red arrow. The time-varying regression coefficients ${\hat{\beta }}_{1}\left(t\right)$ (Fig. 1a) and ${\hat{\beta }}_{3}\left(t\right)$ (Fig. 1b) show a slight abnormality at the starting time point due to the end-point effect of the asymmetrical weighting function with dual optimal bandwidths.

**Fig. 3:**
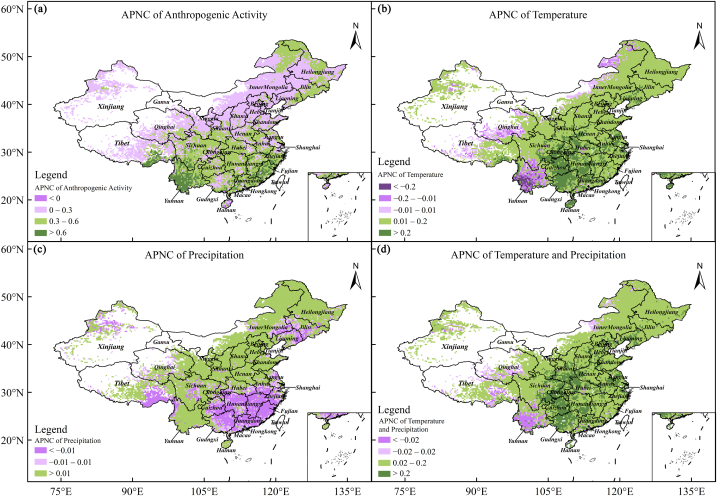
Average predicted contributions of climate and anthropogenic activity to NDVI.

**Fig. 4:**
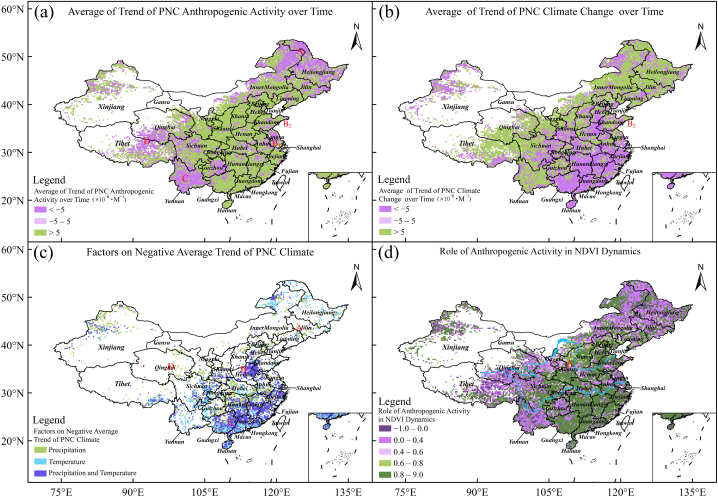
Role of anthropogenic activity in NDVI dynamics. In Panel (d), cyan curved lines represented the Yellow River (upper) and the Yangtze River (down)

### 4.2 Fundamental and derived datasets in the LWLR model

#### 4.2.1 Fundamental datasets

Fundamental datasets primarily refer to NDVI, temperature, and precipitation. Fig. 2a–c shows the fundamental datasets of the LWLR model. The time series of monthly NDVI from January 2000 to December 2019 at each grid point was decomposed into the trend, seasonal, and remainder components using the STL procedure [38]. After the trend term of monthly NDVI was obtained, the monthly slope of the trend term was estimated using the OLS method. Fig. 2a shows the monthly slope of the trend term, which was spatially clustered and heterogeneous. Approximately 13.5% of all grid points had a negative monthly slope (<−2 × 10^−5^ × month^−1^), indicating that the vegetation was degraded. Nearly 5.5% of all grid points had approximate-zero monthly slope (∈[−2, 2] × 10^−5^ × month^−1^), suggesting that the change in vegetation growth was non-significant. Approximately 81.0% of all grid points had a substantial and positive monthly slope (>2 × 10^−5^ × month^−1^), meaning indicating that the vegetation was promoted. Nearly 51.4% of all grid points with the greatest monthly slope (>2 × 10^−4^ × month^−1^) were predominantly distributed in the North China Plain, East China, South China, and their surrounding areas. Meanwhile, grid points with substantial and negative monthly slopes (<−2 × 10^−5^ × month^−1^) were predominantly located in the western Sichuan Basin, Yunnan, the Qinghai-Tibet Plateau (QTP), Jiangsu, southern Hebei, and economically developed urban areas. This finding was consistent with that of Jin et al. [7] but disagreed with that of Geng et al. [49] because the NDVI data source in the latter study was SPOTVGT NDVI, which was different from that in this study.

The respective linear changing trend (monthly slope) for the temperature and precipitation time series at each grid point is mapped in Fig. 2b and c. Overall, the monthly temperature slope for almost all grid points was positive, indicating that atmospheric temperatures are rising. This finding was consistent with current knowledge of global warming [50]. The monthly precipitation slope had a relatively complex spatial distribution. The negative precipitation slope was mainly observed in Tibet, Henan, southwestern Shandong, and northern Anhui. In contrast, a positive precipitation slope was primarily concentrated in southern and Northeast China.

#### 4.2.2 Derived datasets

Derived datasets primarily included the ratio of the dual optimal bandwidths and the respective average of time-varying regression coefficients of temperature and precipitation (Eqs. (19), (20))). Fig. 2d shows the ratio of dual optimal bandwidths (*R*_h_ = *h*_2_/*h*_1_, Eq. (11)) in the LWLR model. The ratio was between 0.9 and 1.1 for approximately 36.5% of all grid points and was greater than 1.1 for approximately 34.4% of all grid points. The grid points with a ratio between 0.9 and 1.1 were distributed in the subtropical humid regions (Jiangsu, Anhui, Henan, Zhejiang, Jiangxi, Fujian, Hunan, and Sichuan Basin) and Northeast China. The small *R*_h_ value was in northern China, where the cases of climatic factors temporally lagging behind NDVI were dominant. This was consistent with other studies, which have demonstrated that precipitation had a 1–3 month time lag behind NDVI in northern China [51]. The greater *R*_h_ was seen in QTP, indicating that NDVI temporally lagged behind climatic factors. This finding was in agreement with Refs. [36,52].

Fig. 2e shows the average of the time-varying regression coefficient of temperature (Eq. (19)). Overall, this coefficient was positive in most of China but negative in Yunnan. The grid points with the greater average were distributed in the eastern QTP, northern Guangxi, and southeastern Guizhou, indicating that rising temperatures increased NDVI. However, the opposite occurred in Yunnan. Yunnan is a typical plateau region [53], dominated by a subtropical monsoon climate [54]. The NDVI is lowest in March but highest in November. The temporal change in temperature is asynchronous with that in NDVI at a monthly timescale, resulting in a negative correlation between NDVI and temperature. Hence, the asynchronous temporal changes in temperature and NDVI caused the negative time-varying regression coefficients of temperature.

Fig. 2f shows the average of the time-varying regression coefficient of precipitation (Eq. (20)), which has a relatively complex spatial distribution. The positive averages were mainly observed in the arid and semi-arid regions of northern and Northeast China, where sufficient precipitation is conducive to vegetation growth. However, the negative averages were primarily observed in the three areas, including parts of southern China (Zhejiang, Fujian, Jiangxi, Hunan, Guizhou, Guangxi, and Guangdong), the southern QTP, and the border areas between Jilin, Liaoning, and Inner Mongolia, where the average annual precipitation is relatively high overall. Therefore, precipitation there is negatively correlated with NDVI, according to Geng et al. [49].

### 4.3 APNC of climate change and anthropogenic activity to NDVI

The APNC of climate change and anthropogenic activity to NDVI was calculated at each grid point across China using Eqs. (13), (14), (15)). Fig. 3 shows their spatial distributions. Fig. 3a shows the APNC from anthropogenic activity. All grid points had positive APNC values, except several in Hainan. The APNC shows a spatial gradient distribution, gradually decreasing from the south to north. The grid points with APNC ∈ (0.01, 0.30), accounting for approximately 54.2% of all grid points, were predominantly situated in parts of Northeast China, Inner Mongolia, the Loess Plateau, Shanxi, Hebei, Shandong, and QTP. Grid points with APNC ∈(0.31, 0.60), accounting for approximately 38.3%, were scattered in Sichuan Basin, Central China, East China, and South China. Grid points with APNC>0.60 accounted for approximately 7.5%, scattered in Fujian, Guangdong, and Yunnan.

Fig. 3b shows the temperature APNC. The positive (>0.01), insignificant (∈[−0.01, 0.01]), and negative (<−0.01) APNC accounted for 86.2%, 7.8%, and 6.0% of all grid points, respectively. The grid points with greater APNC (>0.2), accounting for 15.7%, were primarily scattered across Guangxi, Guizhou, Chongqing, Hunan, and the eastern Sichuan Basin. However, those with negative APNC (<−0.02) were predominantly concentrated in Yunnan.

Fig. 3c displays the precipitation APNC, which was more complex than that of temperature APNC. The amplitude of the precipitation APNC was lower than that of temperature APNC. Only a few grid points with insignificant precipitation APNC ∈[−0.01, 0.01] accounted for 14.2% of all grid points, primarily distributed in Jiangsu and northern Zhejiang. The grid points with positive precipitation APNC (>0.01), accounting for 66.6%, were primarily distributed in North China, Northeast China, eastern Inner Mongolia, and certain regions of QTP, where it was characteristic of aridity, semi-aridity, and semi-humidity. The grid points with negative precipitation APNC (<−0.01) were scattered in Guangxi, Fujian, Hunan, Jiangxi, Guangdong, and the southeastern edge of Tibet, where the annual precipitation is relatively high.

Fig. 3d maps the sum of the temperature and precipitation APNC, that is, the APNC of climate change. Compared with Fig. 3a, the APNC of climate change was smaller than that of anthropogenic activity. We calculated *R*_ac_ = $\frac{{\bar{\beta }}_{0}}{\left(\bar{f}+\bar{g}+{\bar{\beta }}_{0}\right)}$ for each grid point. As a result, the number of grid points with *R*_ac_ < 0.5, *R*_ac_∈(0.51, 0.75), *R*_ac_∈(0.76.1), and *R*_ac_ > 1 accounted for 9.6%, 55.0%, 30.1%, and 5.3% of all grid points, respectively. Hence, most grid points were inferred to be driven by climate change and anthropogenic activity, and the APNC of anthropogenic activity was dominant.

Summarily, the APNC of anthropogenic activity was the highest, followed by temperature and precipitation. The temperature APNC was positive across most of China but negative in Yunnan. The precipitation APNC was positive in the north of the Yangtze River, where precipitation is insufficient; however, it was negative in South China, where precipitation is plentiful.

### 4.4 Relative roles of anthropogenic activity and climate change in NDVI dynamics

#### 4.4.1 Average changing trend of the PNC of anthropogenic activity

The relative roles of climate change and anthropogenic activity in NDVI dynamics were measured by the changing trends of PNC (Eqs. (16), (17))). Fig. 4a shows the average changing trend of the PNC of anthropogenic activity, *α*_0_, which was spatially heterogeneous. The grid points with negative *α*_0_ (<−5 × 10^−6^ × month^−1^), insignificant *α*_0_ (∈ [−5, 5] × 10^−6^ × month^−1^), and positive *α*_0_ (>5 × 10^−6^ × month^−1^) accounted for approximately 31.0%, 4.3%, and 64.7% of all grid points, respectively. The positive *α*_0_ indicated that anthropogenic activity positively contributed to NDVI over time overall and *vice versa*. The positive *α*_0_ was seen in most regions of China. However, the grid points with negative *α*_0_ were predominantly distributed in Northeast China, central QTP, the middle and lower reaches of the Yangtze River (Jiangsu), and the Karst region (Yunnan and Guizhou).

#### 4.4.2 Average changing trend of the PNC of climate change

The average changing trend of PNC of climate change *α* was affected by six factors (Eq. (18)), including the time-varying regression coefficients of ${\hat{\beta }}_{1}\left(t\right)$ (Eq. (19)) and ${\hat{\beta }}_{3}\left(t\right)$ (Eq. (20)), their changing trends $\frac{d{\hat{\beta }}_{1}\left(t\right)}{dt}$ (Eq. (21)) and $\frac{d{\hat{\beta }}_{3}\left(t\right)}{dt}$ (Eq. (22)), temperature, and precipitation*.* Fig. 4b shows the parameter *α,* which was spatially heterogeneous. Approximately 40.0%, 3.7%, and 56.3% of regions in China had negative *α* (<−5 × 10^−6^ × month^−1^), insignificant *α* (∈ [−5, 5] × 10^−6^ × month^−1^), and positive *α* (>5 × 10^−6^ × month^−1^). Grid points with positive *α* were distributed in the regions of Yunnan, Guizhou, QTP, Loess Plateau, Inner Mongolia, and Northeast China. Grid points with negative *α* were distributed in eastern Henan, southern Hebei, Liaoning, and parts of East and South China.

Regarding grid points with negative *α*, the findings in this study were inconsistent with previous studies [7] because monthly timescale datasets were used rather than yearly. Fig. 4c shows the main driving factors on the composition structure of negative *α*, which were temperature, precipitation, and a combination of temperature and precipitation*.* Temperature was the main driving factor in the tropical zone (Guangxi and Guangdong) and the cold temperate zone (Northeast China and Inner Mongolia). The combination of temperature and precipitation was the main driving factor in southern China (Fujian, Zhejiang, Jiangxi, and Hunan), where the rising atmospheric temperature, rich precipitation, and asynchronous temporal changes between precipitation and NDVI [55] have strongly restricted vegetation growth. Precipitation was the main driving factor in the transitional zone between humidity, semi-humidity and semi-aridity [51].

The negative trends of PNC climate change have different mechanisms. The asynchronous temporal changes in NDVI and precipitation caused negative trends in Liaoning (marked “A" in Fig. 4c). This finding was in line with that of [56], who demonstrated that precipitation had a time-lagging relationship with NDVI in Liaoning. Low precipitation and low atmospheric temperature prevented vegetation growth in the QTP (marked “D" in Fig. 4c), which was consistent with [57], who demonstrated that climate warming remains the most noted driving factor for NDVI dynamics in alpine grasslands on the plateau. Drought and asynchronous temporal changes in NDVI and temperature inhibited extensive vegetation growth, leading to the negative changing trends in Henan (marked “B", Fig. 4c). The asynchronous temporal changes in NDVI, temperature, and precipitation impeded extensive vegetation growth in southern China (marked “C" in Fig. 4c). These phenomena (marked “B" and “C" in Fig. 4c) have rarely been reported in the literature.

#### 4.4.3 Relative roles

According to Jin et al. [7], the ratios *R*_a_ = *α*_0_/(*α*_0_ *+ α*) and *R*_c_ = 1− *R*_a_ were defined to represent the relative roles of anthropogenic activity and climate change in NDVI dynamics, termed relative contribution ratios. *R*_a_ < 0 indicated that vegetation growth is degraded owing to anthropogenic activity. Fig. 4d maps *R*_a_. The grid points with *R*_a_ > 0 accounted for approximately 84% regions of China. Among them, the areas with *R*_a_ ∈[0.0, 0.4] and [0.8, 1] were larger, covering approximately 31.3% and 36.4% of the total area in China, respectively. The grid points with *R*_a_ > 0.8 were mainly concentrated in the central Loess Plateau, North China Plain, and South China. However, the grid points with negative *R*_a_ covered approximately 10.1% regions of China, mainly concentrated in Yunnan, QTP, and Northeast China.

The grid points with *R*_c_ > 0 accounted for approximately 56% of the area of China. Among them, the areas with *R*_c_ ∈[0.2, 0.4] and [0.8,1] accounted for approximately 12.5% and 22.8% of the total area, respectively. The grid points with *R*_c_ > 0.8 were mainly concentrated in the northeastern QTP, Yunnan, and Northeast China. However, the grid points with negative *R*_c_ covered approximately 5.5% of the total area, mainly concentrated around the Northeast Plain and in the southeastern QTP. Therefore, based on the average *α*_0_ and *α* over China, we calculate the average nonlinear contribution ratio of anthropogenic activity and climate change to NDVI across China to be approximately 57% and 43%, respectively.

## 5 Discussion

### 5.1 Model

Hydrothermal conditions and anthropogenic activities mainly drive vegetation growth. Monthly datasets represent the vegetation growth within a given growing season. In contrast, yearly datasets represent the vegetation growth of inter-growing season. Pei et al. reported them to be different [58]. According to the linear method (RESTREND) [16], yearly temperature and precipitation contribution rates to NDVI are constant. However, this does not reflect the actual situation because temperature and precipitation have different effects on vegetation growth during the growing period. Therefore, it is appropriate to consider the change in contribution rate over time. High temperatures and drought in a specific year greatly reduce their contribution rates to NDVI.

The Pearson's correlation coefficient between monthly datasets may represent a pseudo-correlation, which does not precisely reflect the impacts of precipitation and temperature on vegetation growth. However, yearly datasets can reduce the possibility of pseudo-correlation by eliminating fluctuations within monthly timescale datasets. Therefore, when Eq. (5) is used based on monthly timescale datasets, whether the residual error passes the normal distribution test is worth examining. If it does not, the autocorrelation or heteroscedasticity of residual error must be removed or reduced using the Cochrane–Orcutt iterative or weighted least squares method.

Using the dual optimal bandwidths, the asymmetric Gaussian-based kernel function could measure the time-lagging relationship between NDVI and meteorological elements. The case of precipitation temporally lagging behind NDVI is likely attributed to the cumulative effect of soil moisture [36,52,59]. Abundant precipitation increased soil moisture and reduced vegetation's dependence on precipitation, resulting in a lagging response of vegetation to precipitation. However, the physical mechanism of NDVI temporally lagging behind precipitation is unclear. We speculated that it is likely attributed to climate response to NDVI change.

### 5.2 Relationship between anthropogenic activity and NDVI

Anthropogenic activities are the key factors that determine the spatial distribution and dynamic change of vegetation. Anthropogenic activities have dual effects on NDVI changes, particularly for crops and grasslands. Modern efficient agricultural engineering plays a significant role in promoting crop growth, and the progress of science and technology over time enhances these roles. Therefore, the contribution rate of anthropogenic activity to NDVI likely varies over time.

Anthropogenic activities positively influence vegetation growth and restoration. The increasing trend of forest and shrub vegetation in Shanxi and Shaanxi is significant (Fig. 4d). The contribution rate of anthropogenic activity to NDVI dynamics was approximately 35% in northern Shaanxi, which was close to the contribution rate (<30%) estimated by Xie et al. [60]. The implementation of ecological protection projects, the returning farmland to forests and grasslands, and the construction of nature reserves helped restore vegetation cover.

However, anthropogenic activities negatively influence vegetation growth. For example, the reduction in APNC of anthropogenic activity in Heilongjiang (marked “A" in Fig. 4a), which was shown by Wang et al. [61], was caused by a great reduction in forest area. From 2008 to 2018, the forest area in Heilongjiang reduced from 2.325 × 10^7^ ha to 2.162 × 10^7^ ha, accounting for approximately 3.40% of the total area under provincial jurisdiction [62]. Piao et al. [63] demonstrated that excessive deforestation and land degradation reduced vegetation cover in Northeast China in recent decades.

Anthropogenic activities severely damaged grassland vegetation in the southwestern Inner Mongolia Plateau and QTP (marked “D" in Fig. 4a). Chen et al. [64] believed that increasing livestock density caused the decrease of aboveground biomass of grassland and the vegetation-cover change in Inner Mongolia, where grazing pressure has not been alleviated but has become ever greater with the increase in fenced grassland areas and livestock density. Furthermore, vegetation growth in the central QTP was affected by grazing/fencing [65]. The influx of tourists and local residents intensified vegetation deterioration after the construction of the Qinghai-Tibet Railway [66]. In addition, the rapid development of animal husbandry in northwestern Sichuan caused harm to the local ecological environment, resulting in a substantial loss of vegetation cover [67]. Therefore, controlling grazing intensity is still a necessary measure for constructing grassland ecological protection in Inner Mongolia and QTP. Additionally, this study observed that the relative contribution ratio of anthropogenic activity gradually declined from east to west in the Yellow River Basin of China (marked “A" in Fig. 4d), which was consistent with Liu et al. [68].

In terms of crops, increasing NDVI is mainly affected by anthropogenic factors. However, anthropogenic damage to crops gradually increased in the vicinity of the Yangtze River Delta region and some provincial capital cities. In Jiangsu (marked “B1” in Fig. 4a), the cultivated land area declines from 4.62 × 10^6^ ha to 4.09 × 10^6^ ha, accounting for approximately 5.2% of the total area under provincial jurisdiction. In contrast, the construction land area increased from 1.75 × 10^6^ ha to 2.09 × 10^6^ ha during 2008–2018 [69]. The extensive conversion of farmland into urban construction land leads to vegetation loss and a decline in NDVI. Therefore, in the strategic context of food security, the negative impact of human activities on crops should receive more attention. Additionally, in the coastal areas of the Shandong Peninsula, although land salinization caused the negative trending of PNC of anthropogenic activity (marked “B2” in Fig. 4a), more precipitation reduced soil salinity to result in a positive trending of PNC of climate change (marked “B2” in Fig. 4b) [70]. The increase in construction land in southern Hebei was the cause of a negatively changing trend in NDVI (marked “B" in Fig. 2a) [71].

Additionally, the Karst regions (Yunnan and Guizhou) experienced negative impacts of human activities on vegetation (marked “C" in Fig. 4a). Fig. 2a demonstrated that the grid points in Yunnan and Guizhou were characterized by vegetation degeneration. These two findings were consistent with each other. Moreover, this result was rarely reported when the linear method (RESTREND) was used [7]. Although nature reserves and safeguard-forest programs have been created in the regions in recent years to prevent rocky soil desertification and promote vegetation growth, the unsustainable utilization of resources has also affected the integrity of regional vegetation.

### 5.3 Relationship between climate change and NDVI

Different precipitation and temperature conditions affect the spatial pattern of vegetation because the response of vegetation growth to changes in heat and soil moisture differs greatly. Rising temperatures positively affect grassland in the southwestern QTP [57]. However, the rising temperature and low annual precipitation intensified aridity trends in arid and semi-arid regions [72]. The degraded vegetation areas driven by climate are distributed in the Junggar Basin, southwestern QTP, and central Inner Mongolia Plateau, which are arid and semi-arid regions. Grassland and desert vegetation are more fragile and have a high risk of vegetation cover degradation. Therefore, the negative effects of climatic factors on vegetation cover should receive more attention.

Additionally, Fig. 2e shows that rising temperatures had non-significant negative impacts on vegetation in Guangdong, which is inconsistent with Huang et al. [73], who showed that in tropical rainforest areas, the average temperature is higher than the optimum temperature for vegetation growth, and further warming is detrimental to vegetation growth. A possible reason for the inconsistency between the two results may be that precipitation was considered in our study, as sufficient precipitation could partially offset the warming effect [74].

### 5.4 Limitations and future directions

The nonlinear contributions from climate and anthropogenic activity to NDVI were estimated using the LWLR model. A few findings were discussed, but there were limitations in the model, data source, and application of the model.

Although the LWLR model can potentially characterize temporal non-stationarity, certain environmental factors (such as altitude) in nature are global. When the LWLR model is applied, there are few false regressions. A mixed temporal-weighted regression model should be used. Additionally, spatial autocorrelation and heterogeneity of natural factors should receive more attention in future studies because autocorrelation and heterogeneity severely impact the results. The selection of appropriate NDVI data is an important prerequisite for study. In previous studies, NDVI products with different time series have been combined to obtain NDVI data with a more extended time series. However, sensors, spectral response functions, and calibration methods for various NDVI products differ, which may influence the results. Furthermore, other natural factors, such as soil moisture, solar radiation, humidity, and soil type, influence vegetation growth. These factors should be considered in the LWLR model in the future. The impact of specific anthropogenic activities, such as vegetation construction, agricultural technology progress, and urban expansion, particularly in the Karst region, on vegetation ecosystem regulation under climate change merits further study. Although these limitations influenced this study, the results still have some reference value for further understanding the dynamic changes of different vegetation types and their driving mechanisms.

## 6 Conclusion

In this study, the nonlinear contributions from climate change and anthropogenic activity to NDVI were quantitatively analyzed using the locally weighted linear regression approach, depending on monthly timescale datasets of NDVI, temperature, and precipitation across China from 2000 to 2019. Their contributions, the changing trends of the contributions, and their relative importance were explored. The main conclusions from this study are as follows.

(1) Vegetation cover exhibited a fluctuation and increasing trend in 81% regions of China from 2000 to 2019. Temperature, precipitation, and anthropogenic activity significantly impacted NDVI. The nonlinear contribution from anthropogenic activity to NDVI was the highest, followed by temperature and precipitation. The spatial distributions of nonlinear contributions from climate change and anthropogenic activity were heterogeneous.
(2) The average predicted nonlinear contribution of anthropogenic activity to NDVI was positive in almost all of China. The temperature APNC was positive in most of China but negative in Yunnan, where the high temperatures and asynchronous temporal changes in temperature and NDVI were the major influencing factors. The precipitation APNC was positive in the north of the Yangtze River, where precipitation is insufficient. However, it was negative in South China, where precipitation is plentiful. Among the extents of the three nonlinear contributions, anthropogenic activity was the highest, followed by temperature and precipitation.
(3)The regions with a relative contribution ratio of anthropogenic activity greater than 80% were mainly distributed in the central Loess Plateau, North China Plain, and South China, while the areas with a relative contribution ratio of climate change greater than 80% were mainly distributed in the northeastern QTP, Yunnan, and Northeast China. The averages of the relative contribution ratios of anthropogenic activity and climate change to NDVI across China were approximately 57% and 43%, respectively.
(4)The average changing trends of climate change and anthropogenic activity contributions to NDVI had highly complex spatial distribution. A negative average of a changing trend in the contribution from climate change was observed in Henan, Liaoning, southern Tibet, Guangdong, Fujian, and Zhejiang. The primary influencing factors were rising atmospheric temperatures, drought, and asynchronous temporal changes in precipitation and NDVI. Among the average changing trends of the contribution from anthropogenic activity, negatively changing trends were observed in central QTP, Yunnan, Jiangsu, and Northeast China, where deforestation, land cover change, and grazing/fencing primarily influenced factors.
(5) Using the dual optimal bandwidths, the asymmetric Gaussian-based kernel function could measure the time-lagging relationship between NDVI and meteorological elements. The NDVI temporally lagging behind precipitation is likely attributed to rich water sources.

## Author contribution statement

Chenhua Shen: Conceived and designed the experiments,; Performed the experiments; Analyzed and interpreted the data; Contributed reagents, materials, analysis tools or data; Wrote the paper.

Rui Wu: Analyzed and interpreted the data; Wrote the paper.

## Data availability statement

Data will be made available on request.

## Declaration of competing interest

The authors declare that they have no known competing financial interests or personal relationships that could have appeared to influence the work reported in this paper.
